# Multiplanar reformation improves identification of the anterolateral ligament with MRI of the knee

**DOI:** 10.1038/s41598-021-92707-w

**Published:** 2021-06-24

**Authors:** Andreas Hecker, Rainer J. Egli, Emanuel F. Liechti, Christiane S. Leibold, Frank M. Klenke

**Affiliations:** 1 Department of Orthopaedic Surgery and Traumatology, Inselspital, Bern University Hospital, University of Bern, Bern, Switzerland; 2 Department of Diagnostic, Interventional and Pediatric Radiology (DIPR), Inselspital, Bern University Hospital, University of Bern, Bern, Switzerland

**Keywords:** Medical research, Ligaments

## Abstract

The anterolateral ligament (ALL) is subject of the current debate concerning rotational stability in case of anterior cruciate ligament (ACL) injuries. Today, reliable anatomical and biomechanical evidence for its existence and course is available. Some radiologic studies claim to be able to identify the ALL on standard coronal plane MRI sections. In the experience of the authors, however, ALL identification on standard MRI sequences frequently fails and is prone to errors. The reason for this mainly lies in the fact, that the entire ALL often cannot be identified on a single MRI image. This study aimed to establish an MRI evaluation protocol improving the visualization of the ALL, using multiplanar reformation (MPR) with the goal to be able to evaluate the ALL on one MRI image. A total of 47 knee MRIs performed due to atraumatic knee pain between 2018 and 2019 without any pathology were analyzed. Identification of the ALL was performed twice by an orthopedic surgeon and a radiologist on standard coronal plane and after MPR. For the latter axial and coronal alignment was obtained with the femoral condyles as a reference. Then the coronal plane was adjusted to the course of the ALL with the lateral epicondyle as proximal reference. Visualization of the ALL was rated as “complete” (continuous ligamentous structure with a tibial and femoral insertion visible on one coronal image), “partial” (only parts of the ALL like the tibial insertion were visible) and “not visible”. The distances of its tibial insertion to the bony joint line, Gerdy’s tubercle and the tip of the fibular head were measured. On standard coronal images the ALL was fully visible in 17/47, partially visible in 27/47, and not visible in 3/47 cases. With MPR the ALL was fully visible in 44/47 and not visible in 3/47 cases. The median distance of its tibial insertion to the bony joint line, Gerdy’s tubercle and the tip of the fibular head were 9, 21 and 25 mm, respectively. The inter- (ICC: 0.612; 0.645; 0.757) and intraobserver (ICC: 0.632; 0.823; 0.857) reliability was good to excellent. Complete visualization of the ALL on a single MRI image is critical for its identification and evaluation. Applying multiplanar reformation achieved reliable full-length visualization of the ALL in 94% of cases. The described MPR technique can be applied easily and fast in clinical routine. It is a reliable tool to improve the assessment of the ALL.

## Introduction

The anterolateral ligament (ALL) is subject of the current debate on rotational stability in association with anterior cruciate ligament (ACL) injuries^1^. Although reliable anatomical and biomechanical evidence for its existence is available today^2^, the impact of the ALL on rotational stability has not been fully understood. Furthermore, the indication to reconstruct the ALL has been discussed controversially^3^.

A biomechanical cadaver study reported abnormal knee kinematics in ALL deficient knees when only the ACL was reconstructed and found normalization of the kinematics if an additional modified deep Lemaire or MacIntosh tenodesis was added^4^. Another study that supports the important role regarding rotatory stability found a significant increase of rotatory instability when the ALL was sectioned additional to an ACL deficiency and therefore, claimed the ALL to be an important secondary stabilizer of the knee^5^. A recent study with a minimum 2 years follow-up was able to link this biomechanical findings to clinical results. They reported a lower reconstruction failure rate and better clinical outcomes in patients without ALL injury compared to patients with ALL injury on the initial MRI. Anterior stability and pivot-shift interestingly did not differ between those two groups^6^. The surgical decision to additionally perform an anterolateral stabilizing procedure is primarily made based on the clinical finding of rotational instability, i.e. a high-grade pivot shift and the type and level of the sporting activity of the patients^7^. An international consensus paper summarized the possible indications for additional anterolateral procedures. They stated that revision ACL reconstruction, high grade pivot-shift, generalized ligamentous laxity, genu recurvatum and young patients returning to pivoting activities may be good candidates for such additional procedures^8^.

The radiological assessment is crucial to distinguish whether rotational instability is due to an ALL injury or another reason such as a meniscal root tear^9,10^. This should ultimately guide the surgical and non-surgical treatment of these patients. For radiologic assessment, an MRI is usually obtained in addition to standard radiographs. Previous studies report ALL identification rates on standard coronal plane MRI images ranging between 11 and 100%^11–13^. A recent review on the identification of the ALL on MRI highlights this heterogeneity and attributes this to different protocols used for the assessment. Moreover, knee sizes plays an important role regarding length, width and thickness of the ALL^14^. This is further supported by a study of patients younger than 18 years, were the authors stated that identification of the ALL was not possible on MRI in females younger than 7 years and males younger than 6 year. In this study a visualization of the ALL in 70% was reached after the age of 13 years in both sexes^15^. Most of the studies reporting on the ALL used standard coronal images for the assessment, while some tried to enhance ALL visualization by slightly flexing and externally rotating the knee during image acquisition^14^.

**Figure 1:**
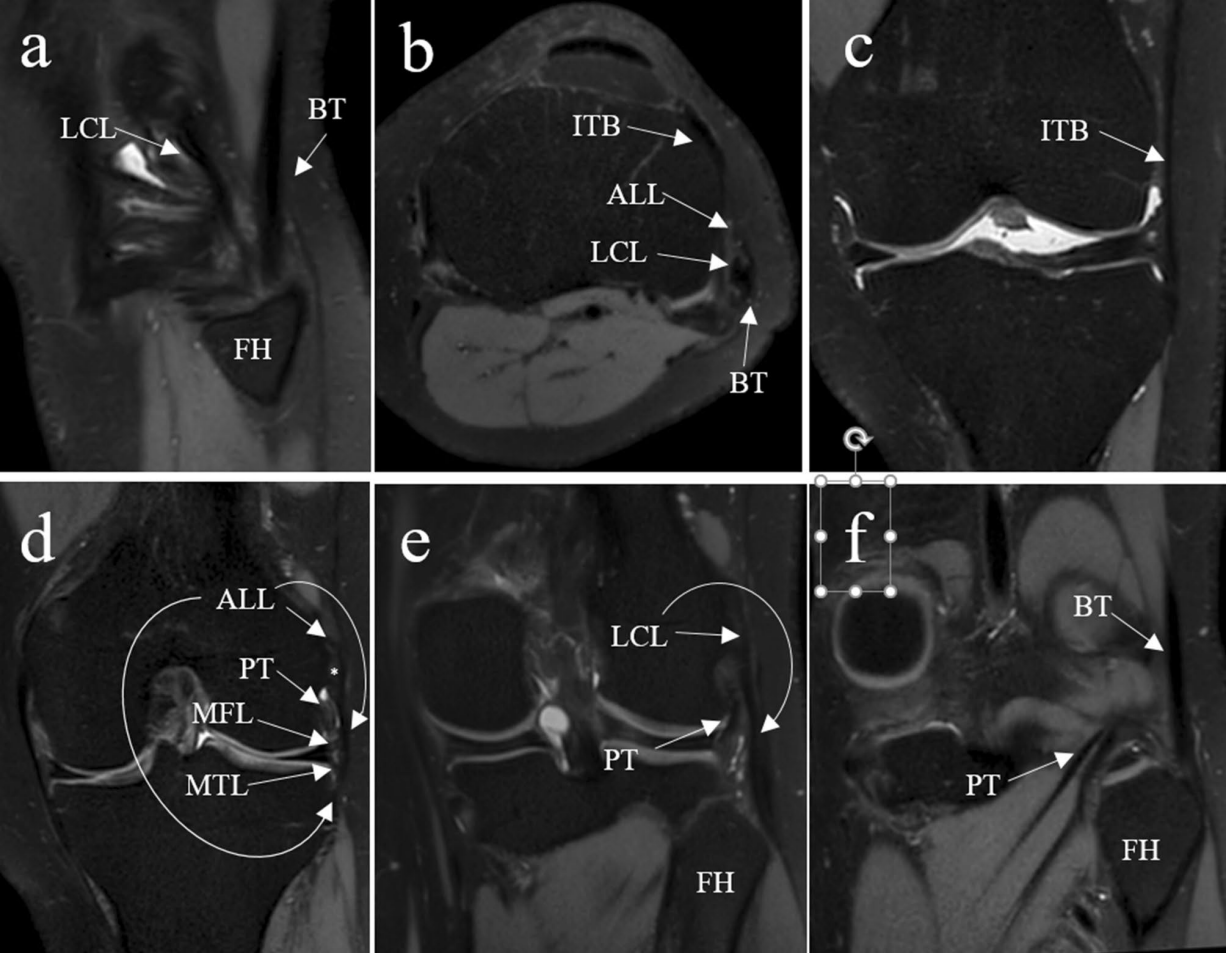
The anterolateral structures are shown in different MRI planes. (**a**) Represents a sagittal MRI image with the lateral collateral ligament (LCL) and the biceps tendon (BT) inserting at the fibular head (FH). (**b**) An axial image that displays the insertion of the iliotibial band (ITB) at Gerdy’s tubercle as well as the anterolateral ligament (ALL), LCL and BT. (**c**) Shows the course of the ITB in a coronal image. In image (**d**) the entire ALL with femoral, tibial and meniscal part is marked as well as the popliteus tendon (PT) and the meniscofemoral (MFL) and meniscotibial ligament (MTL). In this image the lateral epicondyle with the merging fibers of ALL, LCL and MFL is marked with an asterisk. Another coronal image further posterior shows the LCL and the PT (**e**), while in the last image (**f**) even further posterior the BT and PT are shown.

However, identification of the ALL on standard coronal MRI images is challenging and associated with a high level of uncertainty in the discrimination of the ALL and its surrounding structures. This is because the ALL is a fine structure which is usually not aligned precisely to the coronal plane. Furthermore, it merges with the lateral collateral ligament (LCL) near its femoral insertion complicating the discrimination of the two structures proximally. Muramatsu et al. reported 3D-MRI in contrast to the above mentioned 2D analysis allows reliable full length identification of the ALL^16^. To assess the ALL on MRI, a very good anatomical understanding of the anterolateral complex of the knee is required because the capsule, the meniscotibial and meniscofemoral ligaments as well as fibers of the LCL or the iliotibial band may be mistaken as the ALL due to their immediate proximity. Visualization of the whole ALL including its femoral and tibial attachment on one MRI layer seems to be critical for correct identification of the ligament, because if only parts of the ALL are visible, a confusion with other anterolateral structures like the posterior fibers of the iliotibial band, the joint capsule or the meniscotibial and meniscofemoral ligaments is possible. Considering this, a standardized imaging approach using well-defined anatomical landmarks is key but lacking to date. The mentioned anatomic structures are displayed in Fig. 1.

This study aimed to establish an MRI evaluation protocol improving the visualization of the ALL using a standardized multiplanar reformation (MPR) protocol. It was hypothesized that visualization of the ALL can be improved significantly by applying this protocol when compared to the evaluation on standard coronal plane MRI.

## Material and methods

All patients undergoing an MRI performed due to atraumatic knee pain between 2018 and 2019 were retrospectively identified within the picture archiving and communication system (PACS). Only patients analyzed in a 3 T MRI with a 3-dimensional intermediate weighted (proton weighted) fat-suppressed sequence (3D PD VISTA SPAIR; TR 1300 ms, TE 32 ms, slice thickness 0.7 mm) and with a standard coronal reformation (slice thickness 1 mm) of this acquisition directly performed at the MRI console were included. MRIs revealing any injury or pathology as well as previous surgery around the knee were subsequently excluded. Due to technical reasons not anonymized MRI data was analyzed first, the anonymization was done when documenting the measurement results. The study was approved by the local ethics committee and handling of the data was performed in accordance with the guidelines of the Declaration of Helsinki and the Swiss human research act. The ethics committee (Cantonal Research Ethics Commission, Bern, Switzerland) waived the need to obtain informed consent in this study, according to Article 34 of the Swiss human research act.

**Figure 2:**
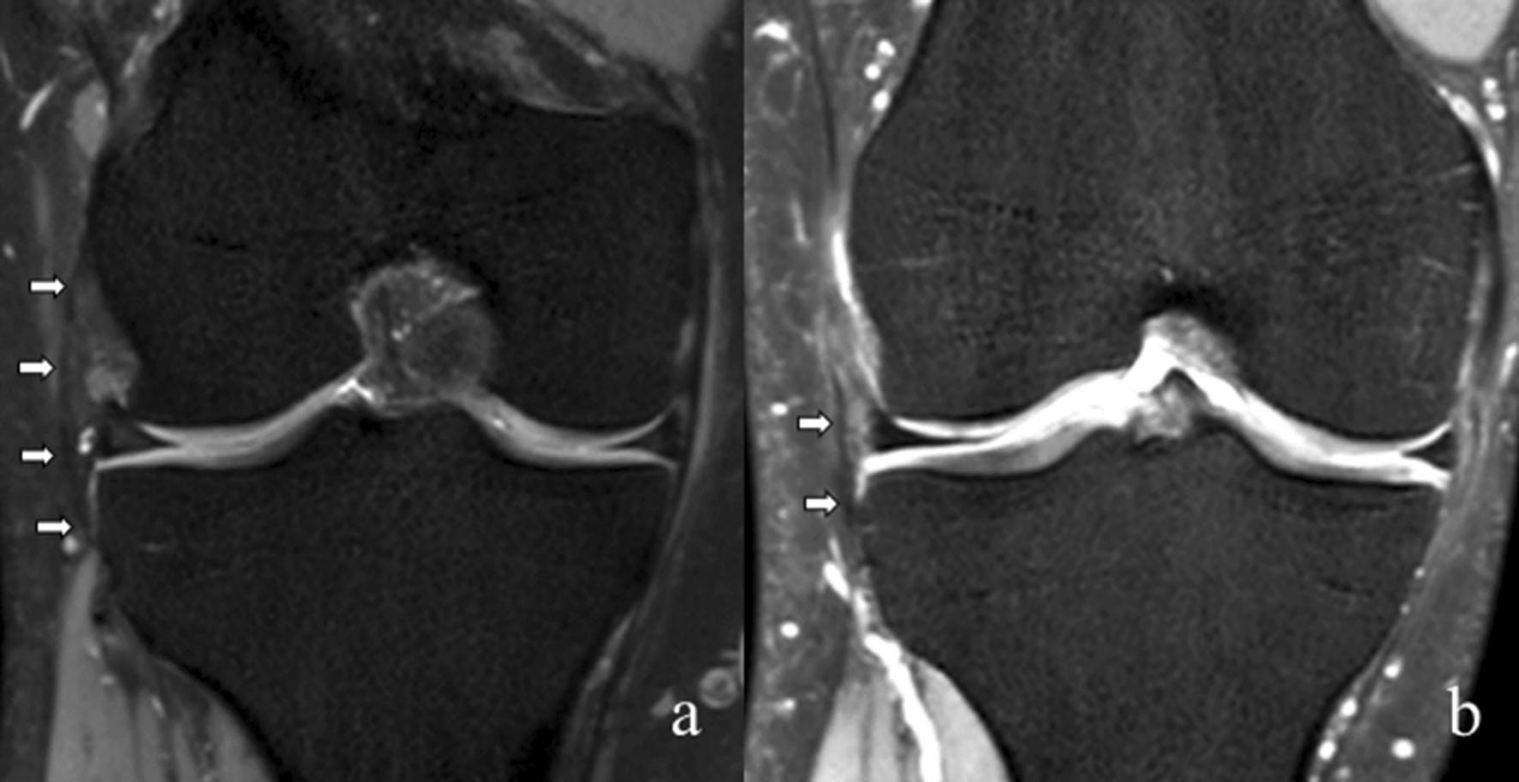
Standard coronal magnetic resonance images of right knees with the anterolateral ligament (ALL) (bold arrows) completely visible (**a**) with femoral and tibial insertion as well as the meniscal portion. The right image shows a partially visible (**b**) ALL.

First, the ALL was identified on standard coronal reformations and classified as completely visible, partially visible and not visible (Fig. 2). Complete visibility was defined as identification of the entire ALL including its tibial and femoral insertions on a single MRI layer. Based on the results of anatomical dissections the femoral insertion was identified at the lateral femoral epicondyle and the tibial attachment midway between Gerdy’s tubercle and the fibular head^17–19^. Second, the 3D acquisition was analyzed using MPR allowing for free orientation of the axial, coronal, and sagittal image planes.

**Figure 3:**
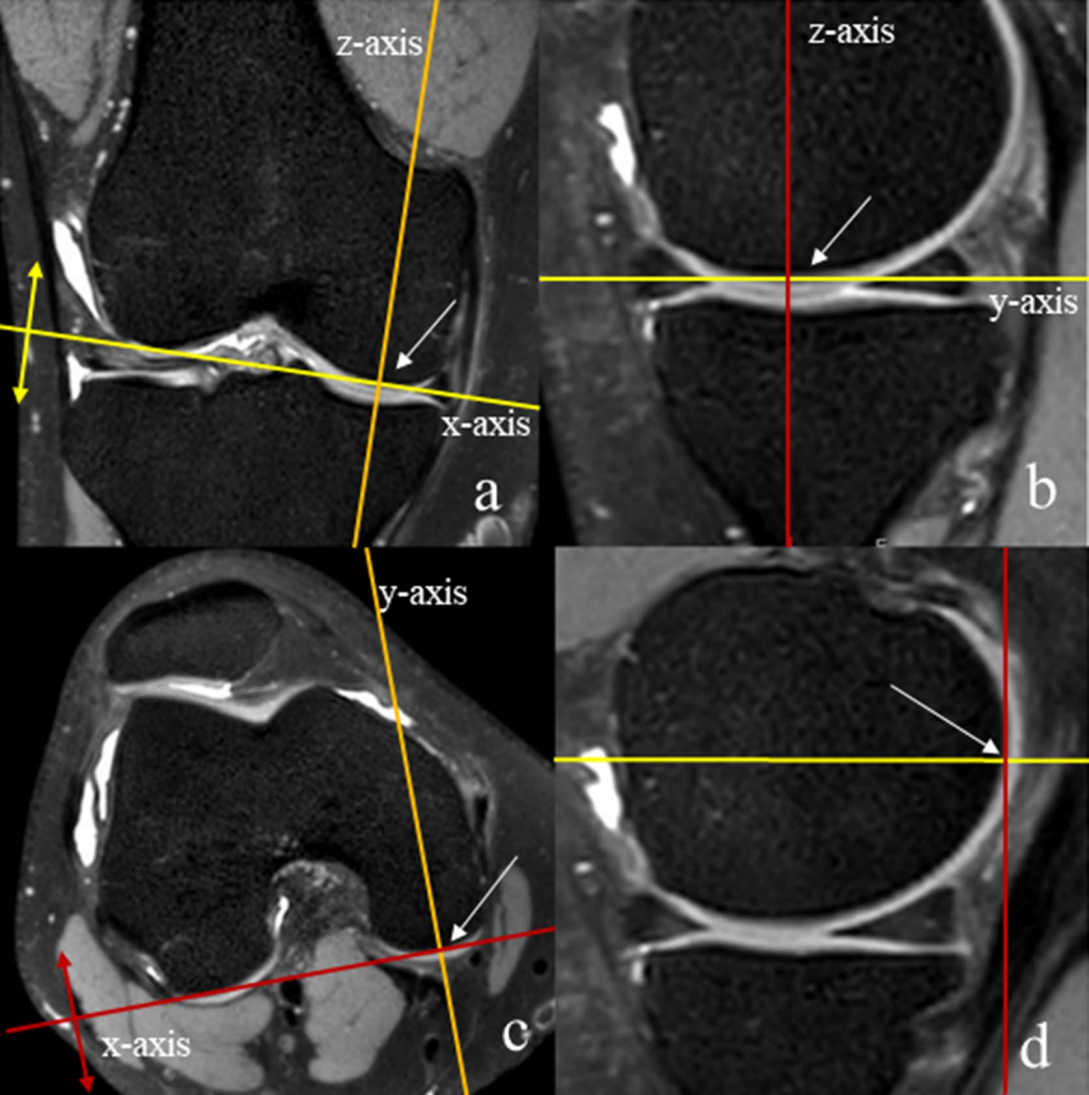
Alignment of the axial plane (**a**,**b**; yellow lines) and the coronal plane (**c**,**d**; red lines). The yellow line representing the axial plane was tangentially aligned to the femoral condyles inferiorly and posteriorly. This alignment was performed in the axial, coronal and sagittal plane. The white arrow marks the center of rotation of the 3D coordinate system.

**Figure 4:**
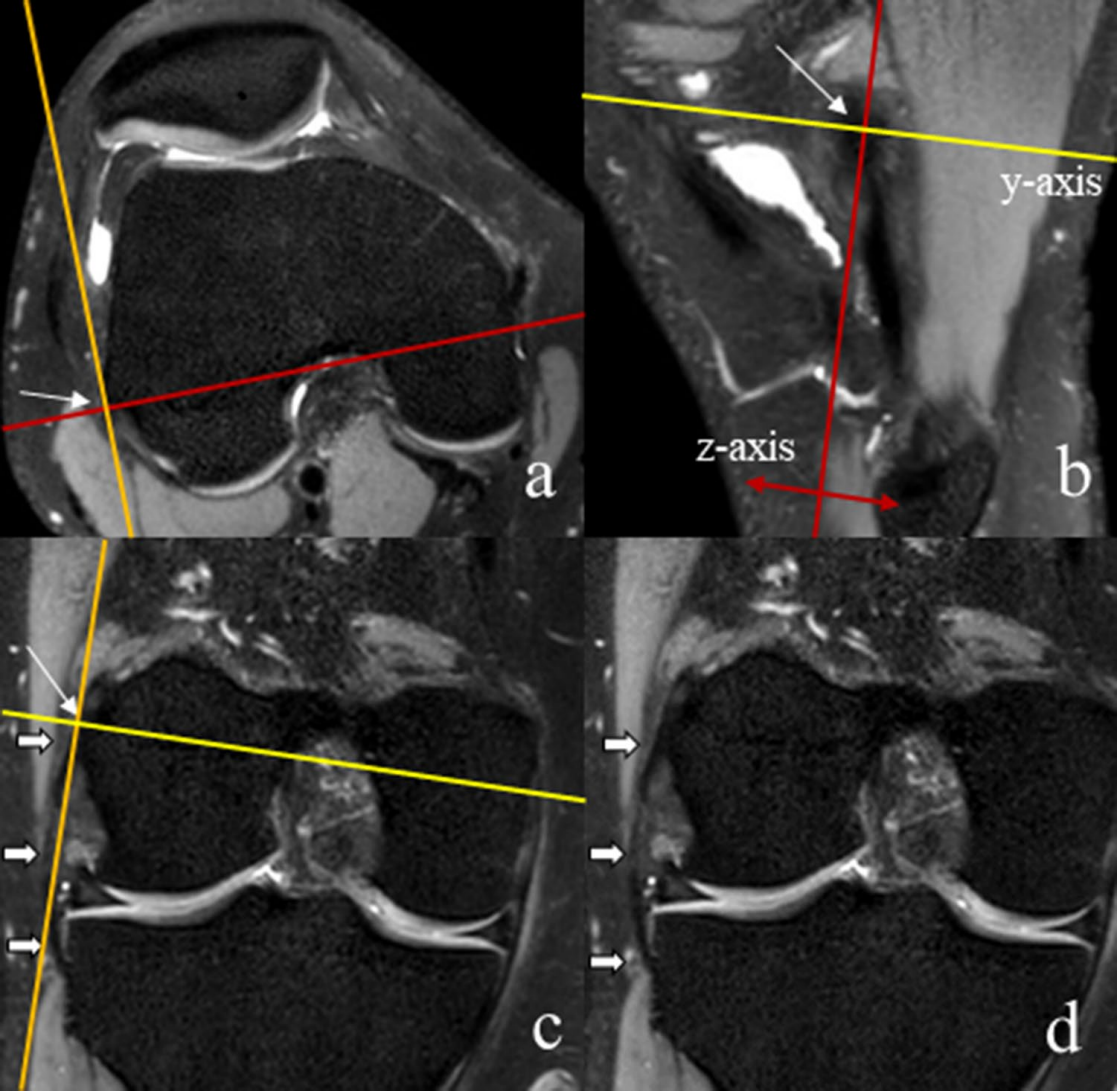
Visualization of the anterolateral ligament (ALL), using multiplanar reformation (MPR) with all planes visible and connected to each other, by tilting the coronal plane in the sagittal image (**b**; z-axis) with the center of rotation at the lateral epicondyle (origin of MPR coordinate system; **a**–**c**; white arrow). This results in full visibility of the ALL in the coronal image (**c**,**d**; white bold arrows). The red arrows indicate the possible directions of tilting the coronal plane around the center of rotation.

To create a reproducible starting position, the axial and coronal planes were aligned to the inferior and posterior edges of the femoral condyles, respectively (Fig. 3). Next, the coronal plane was aligned exactly to the course of the ALL. To this end, the center of the MPR coordinate system was shifted to the proximal and posterior edge of the lateral epicondyle (Fig. 4a), because previous studies reported the proximal insertion of the ALL at or in very close proximity to the lateral epicondyle^19,20^. Then the z-axis was tilted in the sagittal image (Fig. 4b), while observing the lateral ligamentous structures in the coronal image until a continuous structure, the ALL, could be identified from the lateral epicondyle to the tibia between Gerdy’s tubercle (the attachment site of the iliotibial band) and the tip of the fibular head (the attachment site of the collateral ligament) (Fig. 4c,d). Visualization of the ALL was rated as “complete” (continuous ligamentous structure with a tibial and femoral insertion visible on one reformatted coronal image), “partial” (only parts of the ALL like the tibial insertion were visible) and “not visible”. The distances of its tibial insertion to the bony joint line, the midpoint of Gerdy’s tubercle and the midpoint of the tip of the fibular head were measured on coronal and axial images.

All measurements were performed twice by an orthopedic surgeon and a radiologist with a minimum time of 2 weeks between the reads.

SPSS statistics was used for data analysis (IBM SPSS Statistics, Version 25 for Windows). Data was not normally distributed and are given as median and range. Intraclass correlation (ICC) was calculated to assess inter- and intra-observer reliability. Two-way mixed measures checked for consistency and ICC is presented with 95% confidence interval. ICC values of < 0.40 were rated as poor, between 0.40 and 0.59 fair, between 0.60 and 0.74 good and between 0.75 and 1.00 excellent^21^. McNemar’s test was used to compare visibility of the ALL in standard coronal images and after MPR.

### Ethical approval

The study was approved by the local ethical review board (Cantonal Research Ethics Commission Bern, Switzerland) Application BASEC-Nr. 2020-01559.

### Informed consent

Informed consent was not necessary for this retrospective study according to the swiss human research act.

## Results

47 patients could be included for detailed analysis. 28 patients were women and 19 men, the mean age was 29 (range 20–39) and 24/23 were left/right knees, respectively. On standard coronal images Reader 1 rated the ALL as fully visible in 26% (12/47), partially visible in 66% (31/47), and not visible in 9% (4/47) cases in the first read and as fully visible in 28% (13/47), partially visible in 64% (30/47), and not visible in 9% (4/47) cases in the second read (Table 1). Reader 2 rated the ALL on the same images as fully visible in 40% (19/47), partially visible in 55% (26/47), and not visible in 4% (2/47) cases in the first read and as fully visible in 49% (23/47), partially visible in 49% (23/47), and not visible in 2% (1/47) cases in the second read. With MPR Reader 1 rated the ALL as fully visible in 98% (46/47) in both reads and Reader 2 rated the ALL on the same images as fully visible in 87% (41/47) in the first read and in 94% (44/47) in the second read. The rate of full visibility of the ALL improved significantly (p < 0.001) with the application of MRP in both reads of both readers. The median distance of the tibial insertion of the ALL to the bony joint line, Gerdy’s tubercle and the tip of the fibular head was 9 mm (range 6–13), 21 mm (range 11–30) and 25 mm (range 14–34), respectively.

**Table 1:** Summary of the visibility of the ALL on standard coronal and MPR images.

	Reader 1		Reader 2	
	Read 1	Read 2	Read 1	Read2
**Standard coronal images**				
Not visible	4 (9%)	4 (9%)	2 (4%)	1 (2%)
Partially visible	31 (66%)	30 (64%)	26 (55%)	23 (49%)
Completely visible	12 (26%)	13 (28%)	19 (40%)	23 (49%)
**MPR**				
Partially visible	1 (2%)	1 (2%)	6 (13%)	3 (6%)
Completely visible	46 (98%)	46 (98%)	41 (87%)	44 (94%)

The intraobserver reliability for the measurements of the distances of the tibial ALL insertion to the bony joint line, Gerdy’s tubercle and the tip of the fibular head was good to excellent for reader 1 (ICC: 0.720; 0.799; 0.828) and fair to excellent for reader 2 (ICC: 0.544; 0.847; 0.885). The interobserver reliability regarding the same distances was good to excellent (ICC: 0.612; 0.645; 0.757).

## Discussion

This study aimed to establish an MRI evaluation protocol improving the visualization of the ALL using a standardized multiplanar reformation (MPR) protocol. It was hypothesized that visualization of the ALL can be improved significantly by applying this protocol when compared to the evaluation on standard coronal plane MRI. This analysis of 47 knee MRIs shows a significantly better visualization of the ALL after applying the MPR protocol described above, which confirms our hypothesis.

For many years the existence of a distinct ligament in the anterolateral knee capsule called the ALL has been questioned. Today, reliable anatomic, histologic and biomechanical data exist proofing the existence of the ALL^17,22^. More recently, the ALL related debate has shifted towards the clinical relevance of this structure^2^ including controversial discussions on the need for ALL reconstruction when anterior cruciate ligament reconstruction is performed^23,24^. Other possibilities to address the anterolateral rotatory instability are lateral tenodesis of parts of the Iliotibial band with modified Lemaire’s or modified MacIntosh’s techniques being the most referred ones^25,26^. However, there is no clear data available to guide surgical planning yet.

Several authors have claimed to be able to visualize the ALL on standard MRI sequences^10,11,27,28^. After acute ACL injuries Ferretti et al. showed a high prevalence (88%) of ALL abnormalities on MRI, which were significantly associated with lateral joint capsule tears. There were no problems reported in identifying the ALL independent of whether the ALL was injured or not^28^. This is in concordance with many other studies reporting that it is possible to characterize the ALL on MRI in acute case in a high percentage and could also link the MRI findings to the results of surgical exploration during ACL reconstruction^28–30^. Another study investigating the same topic stated that the ALL was not visible in 24% of cases^27^. Helito et al. examined MRI scans of uninjured knees and reported a visibility of the whole ALL in 72% on standard MRI images^12^. A comparative table showing the visualization rates of recent MRI studies about the ALL can be found in the review of Andrade et al.^14^. Porrino et al. stated that in many MRI evaluations different anatomical structures are named as ALL and that a reliable discrimination of the anterolateral structures is very difficult if not impossible on routine MRI^31^. Another drawback of the studies mentioned above is their lack to specify the MRI layer thickness. MRI layer thickness may be a critical factor in identifying the ALL. Large slice thickness can obscure the delicate ALL due to partial volume artifacts/effects by adjacent ligamentous and capsule structures falling within the same voxels during MRI acquisition^32^. In the authors’ opinion it should not be more than 1 mm. This is in agreement with a study by Taneja et al*.* which reported a visibility rate of the ALL of no more than 11% when MRI with a layer thickness of 3 mm were performed^13^.

This overview of MRI studies on the ALL points out the problems associated with its visualization and explains the varying results. The ALL is a very thin structure that may be mistaken easily for the capsule or the meniscotibial and meniscofemoral attachments. Moreover, the ALL lies in very close proximity to the capsulo-osseus layer of the ITB, which is connected to the femur at the intermuscular septum by Kaplan fibers^33,34^. Therefore, on MRI this may give the impression of a continuous structure inserting at the tibia and femur. However, the femoral insertion of these fibers is slightly proximal and posterior to the origin of the ALL, but confusion of these structures on MRI is possible. Therefore, in our opinion, a reliable identification and evaluation can only be ensured, if the whole ALL is visible as a distinct ligamentous structure with clear tibial and femoral attachment on a single MRI image and with a femoral attachment right next to the lateral epicondyle. To reach this goal we applied multiplanar reformation and a novel MRI evaluation protocol based on anatomic landmarks of the ALL. With this technique, the full visibility of the ALL could be improved from 36% on standard coronal MRI images to 94%. However, it must be noted, that even using this technique the visualization of the ALL remains challenging and can only be accomplished by applying the knowledge of anatomical studies.

To be sure that the ALL was identified correctly, we rechecked our results by measuring the distances of its tibial insertion to the bony joint line, the middle of the Gerdy’s tubercle and to the tip of the fibular head. These were found to be 9 mm, 21 mm and 25 mm, respectively, which is within the range of the reported distances in anatomic studies^17–19^. A similar study using a 3D protocol was introduced by Muramatsu et al. in 2018, in this study a reference plane through the lateral epicondyle and a point midway between the posterior edge of Gerdy’s tubercle and the anterior margin of the fibula was chosen. The authors claimed to be able to visualize the ALL in 100% of the uninjured cases using this technique^16^. Our study confirms this findings that the ALL can be visualized in a high percentage using a 3D technique. An advantage of the technique described in our work is, that it primarily uses the femoral condyles to align the MRI in a neutral rotation and neutral varus/valgus and therefore creates a reproducible starting point. Moreover, our reference plane did not go exactly through defined anatomical landmarks, but was oriented exactly to the course of the ALL using those landmarks only as a starting point. With this flexible reference plane the authors tried to address the variations of the femoral and tibial insertions of the ALL.

A major limitation of this study is, that the authors only had access to the MRI data and not to any clinical evaluation or additional demographic data like weight and height. Therefore absolute measurements of the dimensions of the ALL were not reasonable because they are directly linked for example to patient’s height and need to be interpreted in that context. Another limitation is, that the described MPR technique cannot be utilized in standard 3 mm 2D MRI sequences, but needs 3D sequences with maximum 1 mm slice thickness. Moreover, we did not dived the visualization of the ALL in tibial, menical and femoral part as some authors proposed. The reason for this is that many authors claimed that the meniscal and femoral part of the ALL cannot be reliably identified on MRI. Therefor we decided to stay with “partially visible” which is less precise but can be stated more reliable. The goal of this study was to visualize the entire ALL on one MRI image, which could be achieved in 94% of the case using our MPR technique. The question about the parts of the ALL will become more important when the technique described here is used for the assessment of ALL injuries. We will address this in further studies.

## Conclusion

Complete visualization of the ALL is critical for its identification and evaluation. Applying multiplanar reformation achieved reliable full-length visualization of the ALL in 94% of cases. The described MPR technique can be applied easily and fast in clinical routine. It is a reliable tool to improve the assessment of the ALL.
